# Technologies for Type 1 Diabetes and Contact Dermatitis: Therapeutic Tools and Clinical Outcomes in a Cohort of Pediatric Patients

**DOI:** 10.3389/fendo.2022.846137

**Published:** 2022-03-15

**Authors:** Stefano Passanisi, Giuseppina Salzano, Francesca Galletta, Sara Aramnejad, Lucia Caminiti, Giovanni B. Pajno, Fortunato Lombardo

**Affiliations:** ^1^ Department of Human Pathology in Adult and Developmental Age “Gaetano Barresi”, University of Messina, Messina, Italy; ^2^ Department of Human Pathology in Adult and Developmental Age “Gaetano Barresi”, Allergy Unit, University of Messina, Messina, Italy

**Keywords:** allergic contact dermatitis, continuous glucose monitoring, continuous subcutaneous insulin infusion, fluticasone nasal spray, irritant contact dermatitis, skin barriers, topical corticosteroids

## Abstract

The increasing use of technological devices for the management of diabetes is related to the prolonged exposure of patients’ skin to chemical and mechanical agents and, consequently, to the increased risk of developing dermatological complications. Among these, contact dermatitis is the most insidious skin disorder. Despite the magnitude of the issue, no universally accepted recommendations on the management of this common complication are currently available. Our observational study aimed to describe all the solutions adopted by patients and their caregivers to treat and prevent the appearance of contact dermatitis and to describe the clinical impact of this cutaneous complication. Twenty-one pediatric patients (mean age 12.1 ± 3.7 years) with type 1 diabetes were recruited in the study. The most common treatment used to treat acute skin lesions was the application of topical corticosteroids, sometimes associated with topical antibiotics (9.5%). In order to prevent the further appearance of dermatitis, the most frequently adopted measure was the use of hydrocolloid and/or silicone-based adhesives, followed by the application of protective barrier films. One patient reported benefit from the off-label use of fluticasone propionate nasal spray. However, only 52.4% of the study participants achieved a definitive resolution of the skin issue, and 38.1% of patients were forced to discontinue insulin pump therapy and/or continuous glucose monitoring. No differences were observed in glycated hemoglobin values between the period before and after the onset of contact dermatitis. Our study confirms the severity of this dermatological complication that may hinder the spread of new technologies for the management of diabetes. Finally, our findings highlight the importance of establishing close collaboration both with pediatric allergy specialists to prescribe the most suitable treatment and with manufacturing companies to ensure that adhesives of technological devices are free of harmful well-known sensitizers.

## Introduction

Current advanced technologies for the management of type 1 diabetes (T1D) include the following categories: insulin delivery systems, glucose-sensing technologies, and glucose-responsive insulin delivery systems (1). Continuous glucose monitoring (CGM) systems allow patients and providers to monitor current glucose value in real-time, facilitate the achievement of suboptimal glycemic control (2, 3) as well as increase parenteral comfort and decrease fear of hypoglycemia (4). Two types of CGM systems are currently available: real-time CGM (rtCGM) and intermittently scanned CGM (isCGM), also called flash glucose monitoring (FGM). Continuous subcutaneous insulin infusion (CSII) therapy has been demonstrated to decrease intraday glycemic variability and improve psychological outcomes compared with multiple day injection (MDI) (5–7). Furthermore, the most innovative technological devices (i.e. hybrid closed loop and advanced hybrid closed loop), by using an algorithm that automatically modify the basal insulinization rate based on the expected glucose value, allow the achievement of optimal therapeutic goals (8). All these devices are fixed to the skin with an external adhesive patch. CGM systems are approved to be worn for 7–14 days before replacement (1), while CSII infusion sets should be replaced every 2–3 days (9). The extended amount of time of wearing is related to the increased risk of continued, repeated exposure to chemical and mechanical agents. As a result, acute and chronic skin issues may appear and impede comfortable use of these devices (10).

In the last few years, an increase of dermatological complications related to the use of glycemic sensors and/or insulin pumps has been observed. Some recent studies showed that almost 50% of patients using technological devices for the management of diabetes experience skin reactions including eczema, itch, infections, scars, and lipodystrophies under the adhesives of sensors and pump sets (11–15).

**Figure 1:**
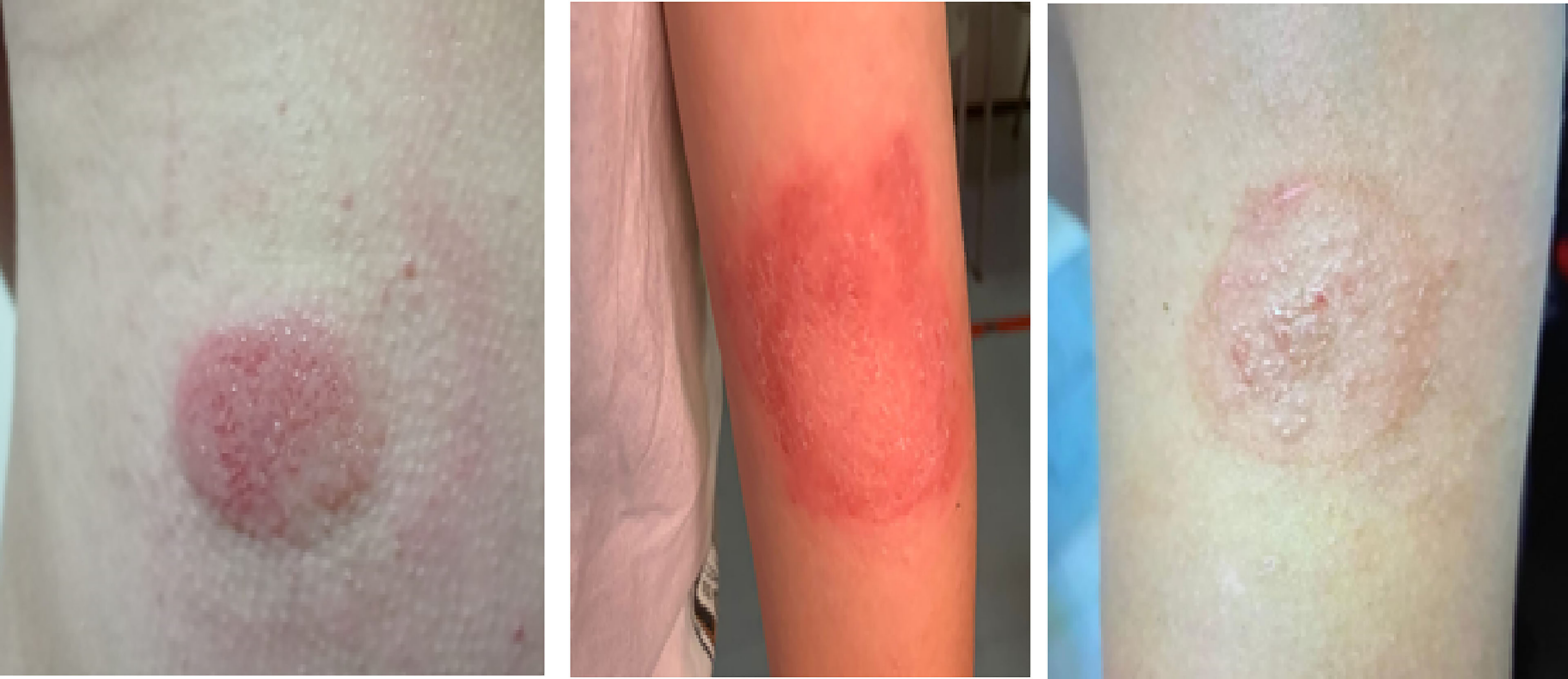
Three cases of contact dermatitis caused by adhesives contained in continuous glucose monitoring devices.

The most insidious among these cutaneous complications is contact dermatitis (**Figure 1**). It is an inflammatory eczematous skin disorder caused by contact irritants that produce irritant effects inducing activation of innate immunity or by contact with sensitizing substances that induce innate and adaptive immune (T-cells) response. Clinical manifestations of contact dermatitis (irritant and allergic) may include erythema, burning, itching, stinging, bleeding and pain (16). In patients with diabetes, contact dermatitis can be caused by the exposure of the skin to potentially harmful chemicals included in the adhesives, plastic catheters and housings of diabetes technological devices (17). This dermatological complication has both a clinical and psychological impact as it affects diabetes-specific emotional distress, leading to a worsening of patients’ quality of life (18).

Despite the increasing number of both adults and children with T1D who presented skin complications, there are few data regarding the clinical impact on the management of diabetes caused by the occurrence of contact dermatitis. Furthermore, no universally accepted recommendations on the management of this common complication are available thus far.

The aim of our monocentric retrospective observational study was to describe all the solutions adopted by patients and their caregivers to treat and prevent the appearance of skin manifestations typical of contact dermatitis. Secondary aim was to evaluate dermatological and glycometabolic outcomes.

## Methods

Our study included children and adolescents (aged 0-18 years) with T1D followed at our Pediatric Diabetes Center, which is the only recognized reference center in the Messina district for diagnosis, treatment and follow-up of youth-onset diabetes. Each patient, or alternatively one of the two parents if a minor, provided their informed consent. The study was approved by the local Ethics committee and conducted in accordance with the Helsinki declaration. The only inclusion criteria for the study was the presence of clinical history positive for skin reactions suggestive of contact dermatitis due to insulin pumps and/or glycemic sensors. The exclusion criteria were the presence of partial clinical remission according to the Hvidovre Study Group definition during the entire study period (19), and the use of measures aimed to treat or prevent contact dermatitis <3 months. Anamnestic data included demographic characteristics (age, sex, race), diabetes duration, presence of atopic comorbidities, insulin treatment type, duration of the use of insulin pumps, FGM or CGM, brand and model of insulin insertion sets and/or glycemic sensors, timing of appearance of skin reactions. All the participants undertook a physical examination with particular attention to skin integrity. Patch testing with specific allergens belonging to resin and acrylate classes were carried out. Acute and preventive treatments were prescribed on the basis of each patient’s clinical history (e.g. results of patch test, type and severity of contact dermatitis), and according to the clinical experience of pediatric allergy specialists of our Department. To evaluate the impact of contact dermatitis on glycemic control, the one-year mean values of glycated hemoglobin (HbA1c) before and after the appearance of skin lesions were compared using the Wilcoxon-signed rank test. Quantitative variables were described using mean and standard deviation. Categorical variables were described as absolute frequencies and percentages. Statistical analyses were performed using IBM SPSS Statistics for Windows, Version 22 (Armonk, NY, IBM Corp.). The significance threshold was set up to 0.05.

## Results

Out of 252 patients with T1D using technological devices and followed at our Pediatric Diabetes Center, 21 (61.9% males) were recruited for the study. Demographic and clinical characteristics of our study cohort are included in **Table 1**. Mean age of the study population was 12.1 ± 3.7 (range 7-18) years and mean duration of diabetes was 6.4 ± 3.3 (range 3-18) years. Atopic history was present in 47.6% of our patients. Patch test was positive in 12 patients (57.1%). More than half the patients had early onset of contact dermatitis, within 3 months of starting use of the patch pump and/or glycemic sensor.

**Table 1:** Anamnestic and clinical data of our study cohort.

Age (years)	12.1 ± 3.7
**Gender**	
Male	13 (61.9%)
Female	8 (38.1%)
Diabetes duration (years)	6.4 ± 3.3
**Atopic predisposition**	
Yes	10 (47.6%)
No	11 (52.4%)
Age at the onset of contact dermatitis (years)	9.2 ± 3.4
**Time of appearance of contact dermatitis**	
0-3 months	12 (57.1%)
3-6 months	1 (4.8%)
6-12 months	2 (9.5%)
>12 months	6 (28.8%)
**Patch test**	
Positive	12 (57.1%)
Negative	9 (42.9%)

Skin issues were mainly present in subjects wearing Enlite^®^ sensor (16.7% of total users). **Table 2** summarizes the relationship between the appearance of contact dermatitis and the total number of patients using different technological devices followed in our Diabetes Centre.

**Table 2:** Relationship between contact dermatitis and the total number of patients using technological devices and followed in our Diabetes Centre.

Device for diabetes management	Total users	Frequency of skin reactions
Medtronic® insulin pump	92	10 (10.9%)
Enlite® glycemic sensor	90	15 (16.7%)
Omnipod® insulin pump	36	3 (8.3%)
Libre® glycemic sensor	54	5 (9.3%)
Dexcom® glycemic sensor	110	4 (3.6%)

Some patients wore more than one device and experienced skin reactions due to different brands of glycemic sensors and/or insulin pumps.

The most common treatment used to treat acute skin lesions was the application of topical corticosteroids (57.1%), sometimes associated with topical antibiotics (9.5%). Some patients used soothing/emollient creams (23.8%) and more rarely topical antihistamines (9.5%).

To prevent the occurrence of further skin reactions, about 57% of patients used hydrocolloid and/or silicone-based plasters, such as Eurofix® (Eurofarm, Belpasso, Italy) and Suprasorb® H (Lohmann & Rauscher GmbH & Co., Neuwied, Germany) to protect the skin before the application of insulin infusion sets or glycemic sensors. Another recurring solution was the application of protective barrier films, such as Askina® barrier film (B. Braun, Melsungen, Germany), Brava® skin barrier spray (Coloplast, Humlebæk, Denmark), and Cavilon® spray (3M, Saint Paul, Minnesota, United States) to confer a shield against offending agents, associated with the application of supplemental plasters. Finally, one patient used fluticasone propionate nasal spray to preserve skin areas a few minutes before the culprit device insertion. As reported in **Table 3**, clinical responses to these protective tools were heterogeneous. Despite any preventive measures adopted, 47.6% of our study population had a negative dermatological outcome. Consequently, 38.1% of patients were forced to discontinue insulin pump therapy and/or continuous glucose monitoring. Regarding glycemic control, evaluated through analysis of the one-year mean values of HbA1c, no differences were observed between the period before and after the occurrence of contact dermatitis (*p-value* = 0.898) (**Table 3**).

**Table 3:** Therapeutic and preventive measures for the management of contact dermatitis and clinical outcomes.

**Acute treatment for contact dermatitis**	
Topical corticosteroids	12 (57.1%)
Topical corticosteroids + antibiotics	2 (9.5%)
Topical antihistamines	2 (9.5%)
Soothing/emollient creams	5 (23.8%)
**Preventive measures adopted**	
Application of hypoallergenic adhesives	12 (57.1%)
Application of skin barrier spray	6 (28.6%)
Application of hypoallergenic adhesives + skin barrier spray	2 (9.5%)
Use of fluticasone spray	1 (4.8%)
**Dermatological outcomes related to the use of different preventive measures**	
Resolution with hypoallergenic adhesives	7/12 (58.3%)
Resolution with skin barrier spray	3/6 (50%)
Resolution with hypoallergenic adhesives + skin barrier spray	0/2 (0%)
Resolution with fluticasone spray	1/1 (100%)
Resolution with any preventive measures	11/21 (52.4%)
**Discontinuation of CSII and/or CGM systems**	
Yes	8 (38.1%)
No	13 (61.9%)
Last year HbA1c mean value (mmol/mol) before the onset of contact dermatitis	49.7 ± 9.1 **p=0.861**
First year HbA1c mean value (mmol/mol) after the onset of contact dermatitis	49.7 ± 8.3

Results are presented as absolute frequencies and percentages for categorical variables, as well as mean and standard deviation for numerical data.

The bold p-value represents a comparison between the “last year HbA1c mean value before the onset of contact dermatitis” and “first year HbA1c mean value before the onset of contact dermatitis”.

## Discussion

Contact dermatitis can be divided into two subtypes: irritant contact dermatitis (ICD) and allergic contact dermatitis (ACD). ICD is a nonspecific response of the skin to direct chemical damage that releases mediators of inflammation from epidermal cells, while ACD is a delayed, type 4 hypersensitivity reaction to exogenous contact antigens, that induces immunological responses due to the interaction of cytokines and T cells. Although some features (e.g. the timing of onset of the rash, the spread of lesions, patch testing responses) may be helpful to distinguish between ACD and ICD, differential diagnosis is usually hard (20). Nevertheless, these two different subtypes of contact dermatitis are not mutually exclusive as destruction of the skin barrier induced by ICD can increase antigenic exposure and exacerbate the appearance of ACD (21). Patch testing represents the diagnostic gold standard of ACD (22), but sensitivity is approximately 70% (23). The validity of a patch test may be altered by inadequate concentrations of the tested substances (24). Patch testing is useful to define the exact etiologic diagnosis and, thus, to identify the culprit allergens. Several studies have revealed that the allergens most frequently responsible of ACD are isobornyl acrylate and *N-N* dimethylacrylamide which were detected within sensors, such as FreeStyle Libre®, Dexcom® and Enlite®, and Omnipod® insulin pumps (25–30). Another common allergen cause of contact dermatitis is colophonium, contained in the Enlite® sensor and Omnipod® (25, 31, 32). Unfortunately, fully, detailed information on the adhesives used in infusion sets and sensors is rarely available: adhesive manufacturers are often reluctant to disclose their exact composition. Furthermore, in producing these devices, different materials can be mixed together, making it difficult to identify which component contained in the adhesive tapes induces contact allergy. Accurate knowledge of potential allergens is fundamental to minimize the risk of false negatives when performing patch testing. The prevalence of ACD caused by technological devices in T1D patients has not yet been well established. Studies available in the literature have shown heterogeneous rates varying from 5.5 to 8.4% (24, 33, 34).

The choice of the most suitable treatment for acute skin lesions is not easy and varies according to the subtype of contact dermatitis. Most of our study population use topical corticosteroids often associated with local application of antibiotics. Topical corticosteroids represent the gold standard for the treatment of ACD, but their prolonged use can cause epidermal atrophy, damage the skin barrier, and increase sensitivity to irritants (35). According to recent evidence, the first-line treatments of ICD consist of physical protection of skin and protective cream/emollient as prescribed to 23.4% of our patients. The use of topical antihistamines should be reserved for the management of mild skin reactions suggestive of irritant contact urticaria, which is clinically characterized by a typical response to the eliciting dose with wheal, flare, and itching on the skin at the site of contact (22, 36). In some cases, the application of topical antibiotics may be helpful to reduce the risk of bacterial infections (37). Moreover, the use of systemic corticosteroids is needed in the presence of concomitant extensive lesions (22, 36). Therefore, the prescription of acute treatment should be personalized to the patient, and close collaboration with a pediatric allergy team with wide experience in both clinical and diagnostic aspects of contact allergy is desirable (38).

Several tools to prevent the appearance of dermatological complications have recently been put forward. Messer et al. proposed a practical guideline to preserve the skin integrity of diabetic patients who chronically use devices for the management of the disease. The authors focused on the importance of correct device placement, good skincare, careful patch removal, and promoting healing of the skin affected by lesions. In addition, they suggested the use of some techniques to minimize the risk of hypersensitivity reactions (21). Among these, the use of potentially hypoallergenic patches was the most frequently reported in our study. It consists of the application of hydrocolloid and/or silicone-based plasters used to block adhesives from sensors and pumps from direct contact with the skin. Unfortunately, hydrocolloid may contain colophonium-like derivatives, thus they are not indicated in colophonium-sensitized individuals. Liquid or spray barrier films were also commonly used in our study. These products are applied before the insertion of insulin pumps or glycemic sensors and can offer sufficient protection from offending agents contained in adhesives. However, other studies showed that the use of barrier sprays is quite limited and some patients often experience incomplete and transient benefits, especially in cases of contact dermatitis due to glycemic sensor that are worn on the skin for up to 14 days (33). Another interesting preventive solution is the off-label use of fluticasone propionate nasal spray, a steroid commonly used to treat acute rhinitis. Recently, Paret et al. reported the benefits of applying fluticasone propionate spray to the skin lesions of 12 patients with skin disease related to the use of CGM systems. The authors demonstrated that the administration of two puffs of this nasal steroid to the skin area before positioning the glycemic sensor was useful to prevent the occurrence of local irritation or dermatitis. Moreover, no significant metabolic or glycemic deterioration was reported (39). Only one patient of our study cohort used fluticasone propionate nasal spray with satisfactory results. Randomized controlled trials with long-lasting follow-up are awaited to evaluate the effectiveness and safety of this preventive measure.

Regarding glycemic outcomes, no differences in HbA1c values were found between the period before and after the onset of skin lesions. However, this finding does not allow to rule out a potential relationship between contact dermatitis and worse glycemic control. As is known, HbA1c reflects average glucose levels of the previous 2-3 months, but does not identify the magnitude and frequency of glucose variation. Other glucose metrics extracted by analysis of CGM systems (i.e. time within range, time below range, time above range, and coefficient of variation) are currently recognized as appropriate and useful clinical targets that complement HbA1c in the evaluation of glycemic control (40). Unfortunately, these data could not be evaluated as some patients had to discontinue the use of CGM systems due to skin complications. Indeed, the most alarming result of our study is related to the relatively high rate of patients (38%) who were forced to discontinue the use of CSII and/or CGM systems. Despite different preventive measures, the most severe cases of contact dermatitis still remain unresolved and avoiding offending agents contained in the adhesives of technological devices represents the only available therapeutic choice. Therefore, close contact between diabetes specialists and manufacturers should be established to minimize the use of some well-known sensitizers in the adhesives.

In conclusions, contact dermatitis is a fairly common dermatological complication in patients with T1D and it may represent a serious hindrance to the increasing spread of new technologies. Despite the magnitude of the issue, there are no clear, universal recommendations on the most suitable management plan for contact dermatitis caused by the use of diabetes devices. Our study confirms the importance of establishing close collaboration both with pediatric allergy specialists to prescribe the most suitable treatment and with manufacturing companies to ensure that adhesives of technological devices are free of harmful, well-known sensitizers.

## Data Availability Statement

The raw data supporting the conclusions of this article will be made available by the authors, without undue reservation.

## Ethics Statement

The study was exempt from ethical committee approval since it was confined to anonymized and unidentifiable data routinely collected at our Diabetes Centre. Written informed consent to participate in this study was provided by the participants’ legal guardian/next of kin.

## Author Contributions

SP wrote and drafted the paper. GS conceived and designed the study. FG, SA, and LC collected data. GBP and FL contributed to the discussion and reviewed the paper. The paper has been read and approved by all the authors and each author considers that the paper represents their honest work.

## Conflict of Interest

The authors declare that the research was conducted in the absence of any commercial or financial relationships that could be construed as a potential conflict of interest.

## Publisher’s Note

All claims expressed in this article are solely those of the authors and do not necessarily represent those of their affiliated organizations, or those of the publisher, the editors and the reviewers. Any product that may be evaluated in this article, or claim that may be made by its manufacturer, is not guaranteed or endorsed by the publisher.
